# Intra-Examiner and Inter-Examiner Reproducibility of Paraspinal Thermography

**DOI:** 10.1371/journal.pone.0016535

**Published:** 2011-02-11

**Authors:** Matthew McCoy, Ismay Campbell, Pamela Stone, Curtis Fedorchuk, Sameera Wijayawardana, Kirk Easley

**Affiliations:** 1 Division of Clinical Sciences, Life University College of Chiropractic, Marietta, Georgia, United States of America; 2 Marietta, Georgia, United States of America; 3 Kennesaw, Georgia, United States of America; 4 Cumming, Georgia, United States of America; 5 Department of Biostatistics and Bioinformatics, Emory University, Atlanta, Georgia, United States of America; Hospital Vall d'Hebron, Spain

## Abstract

**Objective:**

The objective of this study was to evaluate the intra-examiner and inter-examiner reproducibility of paraspinal thermography using an infrared scanner.

**Materials and Methods:**

The thermal functions of a commercially available infrared scanner (Insight Subluxation Station®) were evaluated for clinical reliability. Two practicing clinicians conducted the measures on 100 subjects. Intra class correlation coefficients (ICCs) and concordance correlation coefficients (CCCs) were calculated from the collected data.

**Results:**

Mean bilateral paraspinal skin temperature was 89.78° F and ranged from 88.77° F to 91.43° F. Intra class correlation coefficients (ICCs) for agreement and consistency ranged from 0.959 to 0.976. Concordance correlation coefficients (CCCs) ranged from 0.783 to 0.859 with tight confidence intervals indicating robust estimates of these quantities.

**Conclusion:**

This study revealed excellent intra-examiner and inter-examiner reproducibility of paraspinal thermography using a commercially available unit.

## Introduction

The purpose of this paper is to report on a study of inter and intra examiner reproducibility of a thermal scanning procedure and to briefly review the literature on paraspinal thermal scanning.

Alterations in skin temperature patterns are thought to be associated with aberrations in the function of the sympathetic nervous system innervating the skin vascular beds [1]. Autonomic nervous system control of the organs, glands, and blood vessels is responsible for thermoregulatory control of the person to the dynamics of the outside world [2]. When the outside environment is cool, the body will attempt to conserve heat, resulting in constriction of the arterioles in the skin. Conversely, when the outside environment is hot, the body seeks to eliminate heat through vasodilation of the arterioles in the skin [2].

Thermal readings have been used to detect right-left differences in paraspinal skin temperature since the 1920's [3]-[5].

The theory is that in a healthy patient, skin temperature patterns will change with thermoregulatory control but remain symmetrical across body regions as the body adapts to the environment [6]. For clinicians managing spinal disorders it is argued that segmental or global distortions result in thermal right-left asymmetries and certain fixed patterns may exist as a result [6].

The clinical value of paraspinal thermal scanning is thought to be in determining the overall degree of autonomic abnormality, and the response of the patient to the intervention [6]-[14].

In the analysis of thermal differentials, we are concerned with two factors - symmetry and pattern. Symmetry refers to the difference in temperature between the left and the right side at like points along the spine. The differences in temperature from side to side are maintained within strict limits in healthy persons [1].

In this regard, Uematsu et al. determined normative values for paraspinal temperature based upon 90 asymptomatic “normal” individuals [1]. The authors stated: “These values can be used as a standard in assessment of sympathetic nerve function, and the degree of asymmetry is a quantifiable indicator of dysfunction. Deviations from the normal values will allow suspicion of neurological pathology to be quantitated and therefore can improve assessment and lead to proper clinical management” [1].

Other more detailed papers have been published that review types of thermographic devices and the development, historical origins, and physiological rationales of thermography in clinical practice [11], [15].

### Literature on Reproducibility

The present data confirms several previous studies that demonstrate the reproducibility of paraspinal thermal scanning.

Several studies on intra- and inter-examiner reproducibility have been conducted on various thermal scanning instruments. Spector et al. scanned twelve subjects at several spinal levels and measured the inter- and intra-examiner reproducibility of an infrared instrument [16]. The reproducibility ranged from .940 to .995. These patients were scanned in the prone, seated and standing positions.

DeBoer et al. used three examiners and twenty four subjects to test the inter- and intra-examiner reproducibility of a handheld, infrared paraspinal instrument [17]. The intra class correlation coefficients for all three examiners was .657 with intra-examiner reproducibility ranging from 0.591 to 0.799 indicating moderate-to-good reproducibility.

Keating et al. found weak levels of inter examiner agreement in their study of the lumbar spine in 25 pain free and 21 symptomatic subjects [18].

Owens et al. studied the inter-examiner and intra-examiner reproducibility of 2 examiners utilizing the Tytron C-3000 handheld thermographic scanner [19]. Using thirty subjects, each examiner scanned the subjects twice with the average time for completion of all four scans taking three minutes. They reported intra class correlation coefficients between 0.91 and 0.98. In addition to the reproducibility issues they reported average temperatures from 35.4°C to 30.0°C with the average temperature changing little between scans suggesting that overall skin temperatures were stable during the procedures. The authors state that their results indicated that, based upon their results, changes in thermal scans are actually due to physiological phenomena as opposed to equipment error. This becomes important from a clinical perspective in attempting to determine the clinical meaningfulness of thermal scan results.

Perdew et al. reviewed the reproducibility of test-retest data from several temperature reading instruments applying each to eight points on the backs of 46 subjects. The reproducibility was found to be generally high in this study [20].

Plaugher et al. studied the intra and inter-examiner reproducibility of a thermocouple paraspinal skin temperature differential instrument using nineteen subjects and two examiners and found intra class correlation coefficients of .27 to .85 [21]. Though in their study, they used the break system of analysis where the examiner looks for deflections of the needle to one side or the other caused by temperature differentials. Thus they had to rely on the examiner's interpretation of an abnormal temperature reading as opposed to a computerized reading of temperature differential. Nevertheless they found acceptable levels of reproducibility for all but one of the observations.

The existing literature on reproducibility of paraspinal thermal scanning, with a couple of notable exceptions, shows good-to-excellent reproducibility for the technique.

### Reproducibility of Analysis

Beyond the reproducibility of the testing procedure itself there is the question of the reproducibility of the interpreter's analysis of the graphs and other data produced by the scans. In one of the first studies to address this issue, Stewart et al. conducted a study attempting to establish a model for a reliable method of analysis of thermal data [10]. Using a computer aided method of comparing graphs utilizing a moving Pearson Product Moment correlation and a moving *t*-test, the investigators found that thermal graphs can be compared over time for changes in temperature deviations at any given location.

In a preliminary study, Owens had two experienced and blinded chiropractors judge the similarity of thermal graphs recorded on successive visits [22]. Using 27 subjects and a total of 76 graphs the percent agreement was 38% with a kappa of .0008. Owens urges the development of a numerical computational method in order to garner increased objectivity of thermal pattern analysis.

Using methods similar to Stewart et al., Owens and Stein report on the development of specialized pattern analysis software that accepts data from thermographic instruments [23]. The software provides tools for manipulation and visualization of two overlapping plots for comparison of thermographic scans taken on different occasions. The authors state that further study is needed to determine what factors and range of values can be used to detect the presence of a pattern and related neurological effects.

Hart and Boone provide a descriptive report of a method of determining patterns within paraspinal skin temperature readings whereby they used a modification of the evaluation described by Stewart et al. [6]. The report by Hart and Boone compared cervical and full spine graphs by creating a template and manually measuring and comparing one graph to another. They determined a total percentage of similarity between any two graphs and the number of areas where two graphs were deviating in the same direction. Using a total of 20 graphs they reported a range of 66.3% to 71% agreement for analysis of full spine graphs and a 67% to 83.6% agreement for analysis of cervical spine graphs [6].

In a recent report, Hart et al. looked at the reproducibility of three methods of computer aided thermal pattern analysis [24]. In their study, three examiners compared two sets of scans from 30 subjects using three methods. Two involved manually aligning the graphs prior to a computer software program calculating the percent similarity and the third was done without manual alignment prior to the calculation of similarity.

The study demonstrated the inter- and intra-examiner reproducibility for manually aligning the graphs to range from 0.791 to 0.987 and also showed that aligning the graphs plays a role in maximizing the percent similarity between graphs.

Hart and others described above have done a good, initial job tackling the issue of interpreter reproducibility and computerized analysis. More work should be encouraged and supported in this fruitful area.

## Methods

### Ethics Statement

This study was conducted following approval of the project and the consent process by the Institutional Review Board of Life University. All subjects provided written informed consent prior to participation.

### Subject Selection

100 University students were recruited by announcements and personal contacts. Other than the willingness and ability to volunteer for the study there were no other specific selection criteria in terms of such things as being pain free or other such qualifications and we did not ask or record information regarding the presence or absence of any symptoms or any other anthropometric data. Subjects were scheduled for scanning based upon availability.

### Data Collection

All thermal scans were acquired using the thermal functions of two Insight Subluxation Stations® software version 7.05. The Insight Rolling Thermal Scanner contains three fixed position non-contact sensors. Each sensor utilizes an infrared detector module with a 8-14 um filter as well as a Fresnel lens focusing system. The software is configured to select two of the three non-contact sensors depending on the size of the patient (adult, child and infant settings). The device additionally measures the position of the scanner to the 0.1” via rotary encoder embedded in the wheel axle as well as an event marker which is utilized by the clinician to mark the anatomical landmarks S1, L1, T1, C2 and left and right C1. The Rolling Thermal Scanner provides real-time simultaneous temperature measurements, as well as wheel position and event marker status, and a real-time graph plots differential temperature vs. travelled distance. The system looks at both radiant and ambient thermal energy.

Each examiner utilized only one of the instruments on each day over a period of four days each, one week apart. Two practicing clinicians (alternatively described as examiners or observers A and B in the manuscript), trained in the use of paraspinal thermal scanning carried out all scans and data collection on all study subjects. Each examiner had approximately five years experience utilizing the Insight Subluxation Station and used it regularly in their clinical practice.

The two examiners, blinded from data collection and analysis, scanned subjects with the use of a handheld rolling infrared thermal scanner along the entire spine from S1 to C1. The examiners were in enclosed rooms immediately adjacent to each other so the subjects could move easily from one room to the other. Each subject was scanned twice by each examiner at one sitting before moving to the next examiner. The time it took to have the subject put on a gown, wait for their turn to enter the room and wait for the examiner to enter them into the database prior to being scanned served as the equilibration period. A study coordinator monitored the flow of subjects and kept track of assigning numbers to each subject.

For purposes of scanning, the subject was placed in the seated position with palms up and feet flat on the floor with instructions to look straight ahead. The thermal scanner was placed at S1. Once the software indicated that there was a stable S1 reading, the examiner pressed a trigger on the hand held scanner and began rolling the thermal scanner up the spine. As the examiner crossed each of L1 and T1 they pressed the trigger again without pausing at those sites. The landmarks identify the endpoints of each region of the spine. In order to identify each of the other 20 segments, the Insight software automatically spreads the data points for the spinal region equally across the segments and uses a method of linear extrapolation to calculate the other individual segmental values. The examiner again pressed the trigger when they reached C2. Individual readings were then collected at left C1 and right C1, which were taken “off axis”. The Insight thermal scanner tracks position with a set of wheels that trails the thermopile sensors. The scanner collects one set of data points each 0.10” or 3.94 data points per centimeter.

### Data Analyses

Of the 100 subjects enrolled in the study, seven subject's data were excluded from analyses because only three scans were performed or they were only scanned by one examiner. Therefore the scans from 93 subjects, including 57 males and 36 females, were used for analyses. Age of subjects ranged from 21 to 60 years of age with a mean age of 32 years.

The data were extracted from the Insight software and transferred to Excel spreadsheets which were then imported into SAS® 9.2 to for statistical analyses [25].

## Results

Statistical comparisons of the paraspinal skin temperature were made across the two examiners and the two repeated measures for each examiner. In Table 1, we report the average left and right paraspinal temperatures and the average bilateral paraspinal temperature for each spinal location. Overall mean bilateral paraspinal skin temperature was found to be 89.78° F and ranged from 88.77° F to 91.43° F with a standard deviation of 0.59° F.

**Table 1:** Overall mean of the bilateral paraspinal skin temperature at each spinal location.

Average Left(n = 400)	Spinal Location	Average Right(n = 400)	Bilateral Average(n = 800)
91.37	C1	91.49	91.43
90.08	C2	90.35	90.21
89.84	C3	90.01	89.92
89.63	C4	89.70	89.66
89.56	C5	89.58	89.57
89.32	C6	89.41	89.37
89.21	C7	89.38	89.30
89.28	T1	89.49	89.39
89.77	T2	90.01	89.89
90.23	T3	90.55	90.39
90.39	T4	90.73	90.56
90.39	T5	90.65	90.52
90.27	T6	90.48	90.37
90.01	T7	90.24	90.12
89.75	T8	90.02	89.88
89.58	T9	89.84	89.71
89.52	T10	89.75	89.64
89.55	T11	89.74	89.65
89.63	T12	89.82	89.72
89.61	L1	89.87	89.74
89.49	L2	89.80	89.65
89.24	L3	89.58	89.41
88.84	L4	89.16	89.00
88.62	L5	88.92	88.77
88.64	S1	88.94	88.79

Overall mean bilateral paraspinal skin temperature was 89.78° F and ranged from 88.77° F to 91.43° F with a standard deviation of 0.59° F.

### Reproducibility Statistics – Intra-Examiner

The Intra class Correlation Coefficients (ICCs) for agreement and consistency were used to test for the reproducibility of the temperature measurements between the two examiners. The ICC is a measure of the amount of overall data variance due to between-subjects variability. ICC (Consistency) emphasizes the association between examiners' scores, while ICC (Agreement) emphasizes the “interchangeability” of the examiners [26]. ICCs are calculated using the variance estimates obtained by modeling the average bilateral skin temperature readings using a mixed effects model.

We use the term bilateral to refer to the average of each examiner's readings from the left and right side of each spinal location for a given trial. The two-way ANOVA model with interaction is defined as:
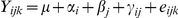
where 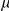 is the overall mean, 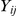 is the average bilateral skin temperature for subject *i* as measured by examiner *j* on the *k* ^th^ trial, 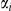 is the random effect of subject 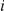, 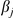 is the fixed effect of observer 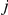, 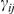 is the random interaction effect and 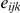 is the random error component.

Let 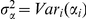, 
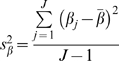
, 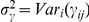 and 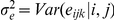, where *J* is the number of examiners.

Note that this model assumes that the error variance, 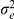, is the same for each subject and observer and that the interaction variance, 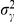, is the same for each examiner.

Using the above statistics, the two ICC's are defined as:
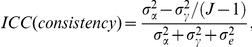


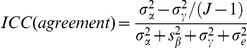



The ICC's are estimated by substituting the estimated variance components into the relevant expression.

The ICCs for agreement ranged from 0.959 to 0.973 and the ICCs for consistency ranged from 0.975 to 0.976 (See Table 2). These numbers suggest excellent intra-examiner reproducibility of paraspinal temperature readings. In other words both examiners on their second trial were able to reproduce their results from the first trial with a high degree of agreement and consistency.

**Table 2:** Intra-examiner Reliability Statistics.

		Left Side		Right Side	
		ICC - Agreement	ICC - Consistency	ICC - Agreement	ICC - Consistency
**Examiner A**	**Trial 1 Vs. Trial 2**	0.9732	0.9756	0.9734	0.9758
**Examiner B**	**Trial 1 Vs. Trial 2**	0.9650	0.9749	0.9586	0.9752

### Reproducibility Statistics – Inter-Examiner

The Concordance Correlation Coefficient (CCC) is based on the distance in the plane of each pair of data to the 45 degree line through the origin and was used to quantify the reproducibility of multiple readings made by the same examiner [27]. CCC's were calculated adjusting for left/right, spinal location and for trial 1/trial 2 differences. These adjustments allow a more accurate assessment of the true variability in each examiner's readings by removing added variability due to these factors from the data.

Following the formulation by Haber et al. [28] the inter-examiner CCC is defined as follows: 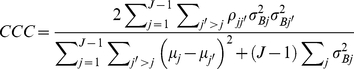
where 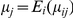, 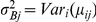, 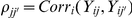 for 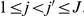


Estimation of these coefficients is made using SAS PROC MIXED, as outlined in Haber et al. [19].

The CCCs ranged from 0.783 to 0.859 with tight 95% confidence intervals indicating that these were robust estimates (See Table 3). The reported CCCs associated with repeat readings were adjusted for left/right and spinal location differences along the spine. Similarly, the reported CCCs associated with left and right spinal temperature measurements were adjusted for repeat readings (trial 1 and trial 2) and spinal location differences. In general, examiner A's left and right, trial 1 and trial 2 measurements showed excellent agreement with the corresponding measurements of examiner B, with a CCC of at least 0.827.

**Table 3:** Inter-examiner Reliability Statistics.

	CCC (95% CI)
**Examiner A Trial 1 Vs. Examiner B Trial 1**	0.846 (0.838, 0.854) *
**Examiner A Trial 2 Vs. Examiner B Trial 2**	0.827 (0.818, 0.835) *
**Examiner A Trial 1 Vs. Examiner B Trial 2**	0.800 (0.790, 0.809) *
**Examiner A Trial 2 Vs. Examiner B Trial 1**	0.859 (0.851, 0.866) *
**Examiner A Left Side Vs. Examiner B Left Side**	0.859 (0.848, 0.869) @
**Examiner A Right Vs. Examiner B Right Side**	0.841 (0.830, 0.852) @
**Examiner A Left Side Vs. Examiner B Right Side**	0.783 (0.768, 0.797) @
**Examiner A Right Side Vs. Examiner B Left Side**	0.845 (0.834, 0.857) @

*- CCC is adjusted for Left/Right difference and differences in Spinal Location.

@ - CCC is adjusted for Trial 1/Trial 2 difference and differences in Spinal Location.

### Coefficient of Inter-Observer Variability

In addition to the commonly reported inter-examiner reproducibility statistic, the ICC, we report the Coefficient of Inter-Observer Variability (CIV), which is a more recent reproducibility statistic that addresses some of the drawbacks associated with using the ICC. The ICC relies on quantifying the total observer variability. However, when the main interest is in true differences among examiners reporting different values of the same quantity, the focus should be more on the inter-observer variability component. Looking at total observer variability masks these sources of disagreement as it contains both inter-observer variability (true differences among examiners) and intra-examiner variability (random error among the observations made by the same observer on the same subject).

The CIV is defined as the ratio of the inter-observer variability to the total observer-related variability. It varies between 0 and 1, and a higher value of the CIV indicates a lower level of inter-observer agreement. If CIV  = 0, then one does not expect any ‘true’ differences among the observers, in the sense that all the observers have the same distribution over the subjects. The quantity 1 - CIV (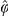) can then serve as a coefficient of inter-observer agreement.

An alternative coefficient related to inter-observer agreement is the Coefficient of Excess Observer Variability (CEOV). This is simply a one-to-one function of the CIV, but is useful in the sense that it provides an alternative and more intuitive interpretation of the CIV.

The estimated CEOV for the inter-examiner variability between left and right measurements in our study is 1.56. This suggests that the total inter-examiner variability between left and right readings is approximately 1.56 times higher than one would expect if the two examiners were perfectly equivalent in their readings of the left and right paraspinal temperatures. The COEV for the total inter-examiner variability between trial 1 and trial 2 readings is 1.32, suggesting that the observed variability is approximately 1.32 times higher than one would expect if the two examiners were perfectly equivalent in their readings (See Table 4).

**Table 4:** Overall Inter-Examiner Variability Coefficients.

Examiners A, B	CIV (95% CI) a	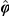 (95% CI) a	CEOV
Left Side Vs. Right Side	0.35977(0.23598, 0.56757)	0.64023(0.43243, 0.76402 )	1.56 *
Trial 1 Vs. Trial 2	0.24432(0.15590, 0.36694 )	0.75568(0.63306, 0.84410 )	1.32 **

**a**– 95% CIs are based on 2000 bootstrap samples.

*The total inter-examiner variability between Left and Right readings is 1.56 times higher than one would expect if the two examiners were perfectly equivalent in their readings.

**The total inter-examiner variability between Trial -1 and Trial-2 readings is 1.32 times higher than one would expect if the two examiners were perfectly equivalent in their readings.

• ***Definition of terms***

○ CIV  =  Coefficient of Inter-Observer Variability.

○ 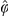 =  Agreement Coefficient based on CIV.

○ CEOV  =  Coefficient of Excess Observer Variability.

The CEOV can range from 1 to infinity. A CEOV of 1 implies that the examiners were perfectly equivalent in their readings. In this sense, coefficients greater but close to one represent better inter-examiner reproducibility compared to values greater than 1. However, given the lack of reproducibility studies on thermal scanning where this statistic was used, we do not have a benchmark range of values, against which we can compare our results.

### Scatter and Bland-Altman Plots

**Figure 1:**
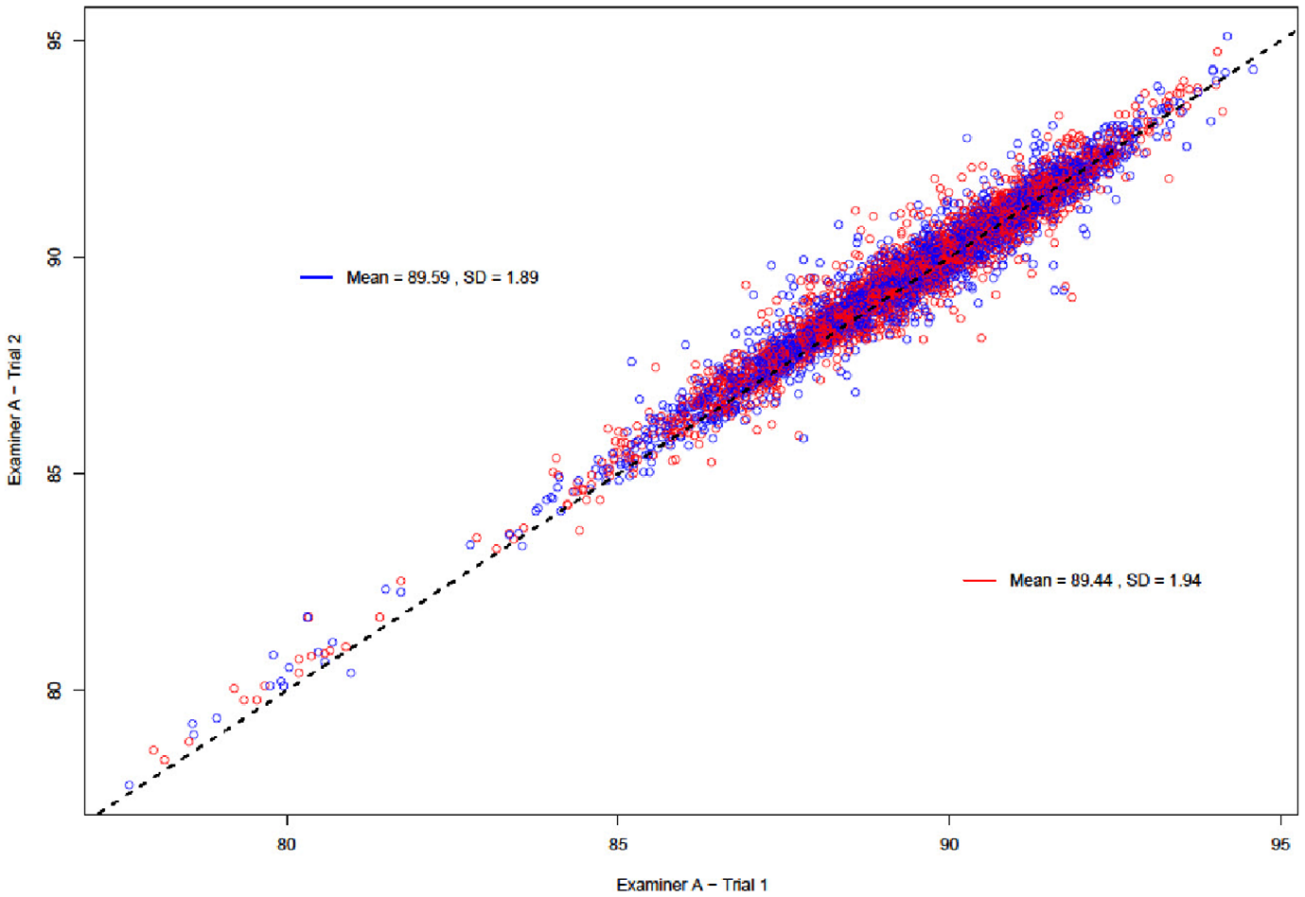
Scatter Plot: Intra-examiner agreement (Examiner A). Figure 1 presents overall scatter of the data corresponding to the intra – examiner agreement for Examiner A. The plot shows a good distribution of data points around the 45° degree line, indicating a high degree of correlation between Examiner A's repeat measurements.

**Figure 2:**
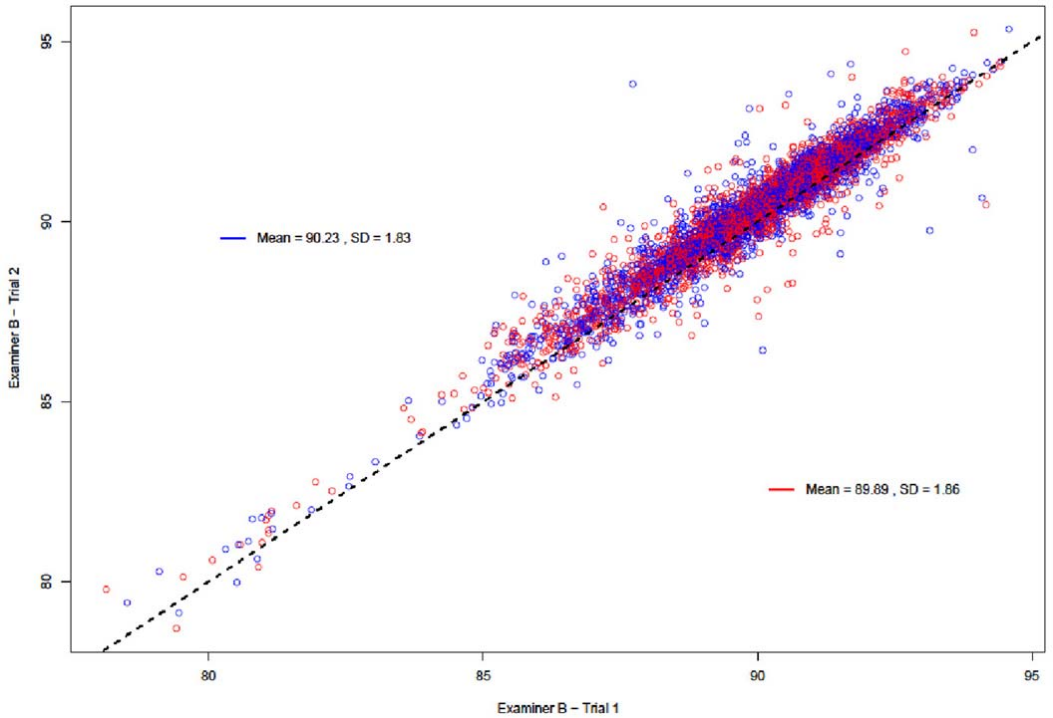
Scatter Plot: Intra-examiner agreement (Examiner B). Figure 2 presents overall scatter of the data corresponding to the intra – examiner agreement for Examiner B. The plot shows a good distribution of data points around the 45° degree line, indicating a high degree of correlation between Examiner B's repeat measurements.

**Figure 3:**
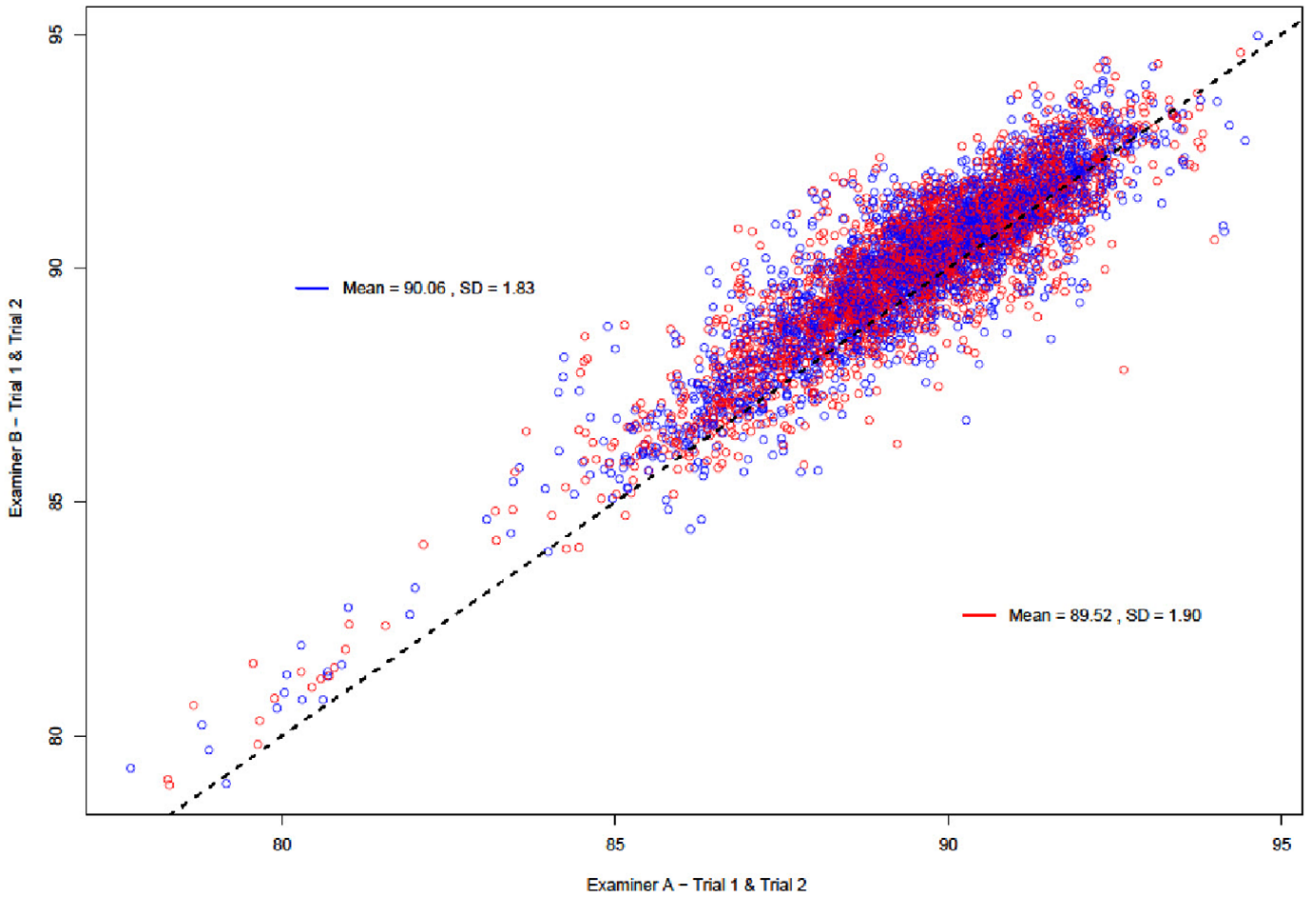
Scatter Plot: Inter-examiner agreement (Examiner A vs. Examiner B). Figure 3 presents overall scatter of the data corresponding to the inter – examiner agreement for both examiners. The plot shows a good distribution of data points around the 45° degree line, indicating a high degree of correlation between the measurements of the two examiners.

Figures 1, 2 and 3 present the overall scatter of the data. Figures 1 and 2 correspond to intra – examiner agreement and Figure 3 corresponds to inter – examiner agreement. All three plots show a good distribution of data points around the 45° degree line, indicating a high degree of correlation between each examiner's repeat measurements and between the measurements of the two examiners.

**Figure 4:**
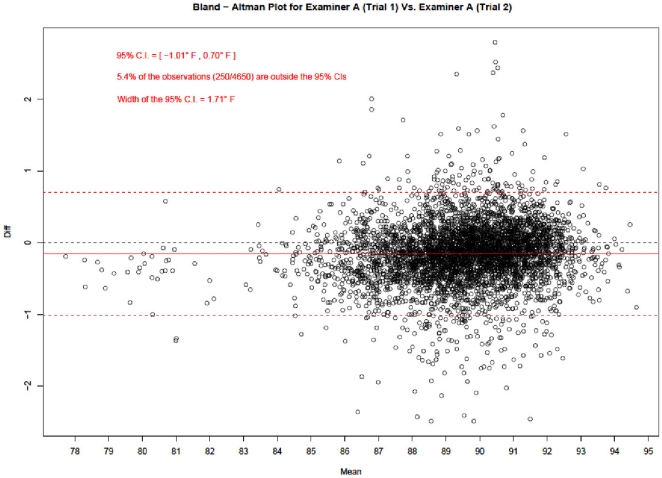
Bland-Altman Plot for Examiner A (Trial 1) vs. Examiner A (Trial 2). Figure 4 corresponds to intra – examiner agreement and shows that only 5.4% of all the readings done by Examiner A fell outside the 95% agreement limits.

**Figure 5:**
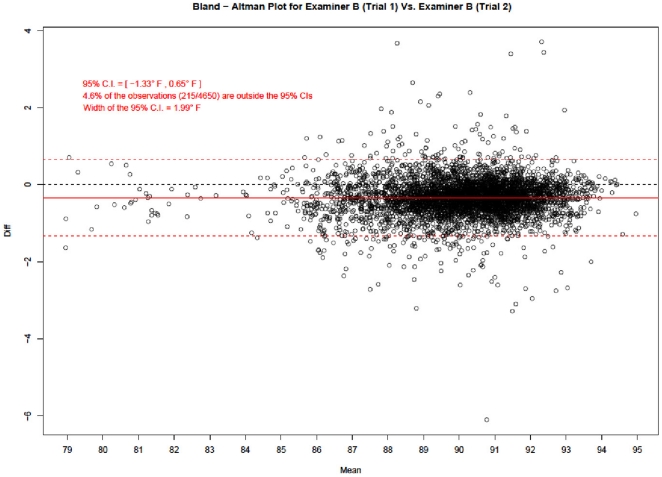
Bland-Altman Plot for Examiner B (Trial 1) vs. Examiner B (Trial 2). Figure 5 corresponds to intra – examiner agreement and shows that only 4.6% of Examiner B's readings fell outside the 95% agreement limits.

**Figure 6:**
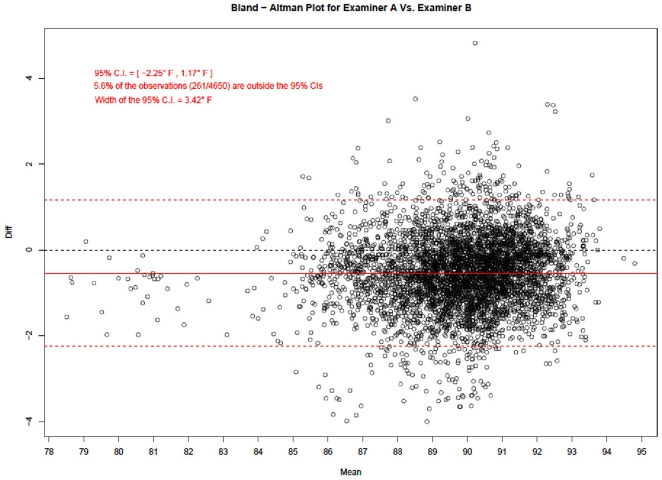
Bland-Altman Plot for Examiner A vs. Examiner B. Figure 6 shows the Bland-Altman plot corresponding to the inter–examiner agreement between the two examiners. On the horizontal axis is the average of the 1^st^ and 2^nd^ temperature readings and on the vertical axis, the difference between the measurements of the two examiners. The plot shows the 95% agreement limits between the measurements of the two examiners with only 5.6% of readings falling outside the 95% agreement limits.

Figures 4, 5 and 6 show the Bland – Altman mean difference plots. A common approach in agreement studies (useful when there are only two examiners) is to calculate the mean of the differences between the two examiners. The confidence limits around the mean provide insight into how much random variation may be influencing the ratings. If the examiners tend to agree, the mean will be near zero. If one examiner is usually higher than the other by a consistent amount, the mean will be far from zero, but the confidence interval will be narrow. If the examiners tend to disagree, but without a consistent pattern of one rating higher than the other, the mean will be near zero but the confidence interval will be wide.

Bland and Altman plots graph the difference of each point, the mean difference, and the confidence limits on the vertical against the average of the two ratings on the horizontal. The resulting plot demonstrates the overall degree of agreement.

In each graph, there are four horizontal lines. The middle red line represents the observed mean difference, and the middle black line represents the expected mean difference under perfect agreement, i.e., zero. The bottom and top red lines represent the 95% limits of agreement. These 95% limits of agreement define the range within which most of the differences between the repeat measurements of each examiner or between the measurements from the two examiners should lie.

Figures 4 and 5 correspond to intra – examiner agreement and Figure 6 to inter – examiner agreement. Figure 4 shows that only 5.4% of all the readings done by examiner A fell outside the 95% agreement limits. Similarly, Figure 5 shows that only 4.6% of examiner B's readings fell outside the 95% agreement limits.

Figure 6 shows the Bland-Altman plot corresponding to the inter–examiner agreement between the two examiners. On the horizontal axis, we've plotted the average of the 1^st^ and 2^nd^ temperature readings and on the vertical axis, the difference between the measurements of the two examiners. The plot shows the 95% agreement limits between the measurements of the two examiners with only 5.6% of readings falling outside the 95% agreement limits.

The fact that approximately 5% of readings fall outside the 95% confidence limits, indirectly validates the normal distribution assumption for our data. This is important since the ICCs are calculated using an ANOVA model that requires the data to be normally distributed.

Not surprisingly, the difference in inter-examiner average readings (width of 95% C.I.  = 3.42°F) is much higher compared to the difference in intra-examiner average readings (widths of 95% C.I.s  = 1.71°F and 1.99°F).

## Discussion

Several studies have found good to excellent reproducibility for paraspinal thermal scanning using a variety of devices. Utilizing the Insight Subluxation Station, our study found excellent inter- and intra- examiner reproducibility for paraspinal thermal scanning using two examiners and the largest number of subjects described in the literature thus far. The Bland – Altman analysis of our data and the Intra class correlation coefficients (ICCs) for agreement and consistency showed excellent intra-examiner agreement. Concordance correlation coefficients for inter-examiner agreement were also high with tight 95% confidence intervals indicating robust estimates of these quantities. The CEOVs for inter-examiner variability between left, right and trial 1, trial 2 were both fairly close to one. A CEOV of one indicates zero variability or perfect inter-examiner agreement.

Given the existence of normative data for paraspinal temperature and the depth of literature demonstrating good to excellent reliability of paraspinal thermal scanning we suggest that investigators move on to explore the clinical meaningfulness of this instrumentation in the management of spinal disorders.

Such explorations should include the relationship between thermal scanning and various spinal disorders. In the case of chiropractic this would include the relationship between vertebral subluxation and articular dysfunction with thermal findings. Research on thermal scanning as an outcome measure and its relationship to health status should be undertaken.

Beyond studying thermal scanning and its relationship to general spinal disorders, its utility in indentifying dysautonomia and suboptimum adaptation responses should also be explored.

### Limitations of the Present Study

Our study focused on inter and intra examiner reliability of a thermal scanning method and instrumentation. We did not intend to address clinical applications nor the sensitivity, specificity and predictive value of thermal scanning in relation to the diagnosis of spinal disorders.

Our study results may have been biased by the long term use of the equipment by the examiners in this study and as such the results may say something about familiarity with the equipment. In either event no definitive statement can be made without comparing data from more inexperienced examiners. If future studies demonstrated that inexperienced examiners produced less reliable scans then training issues would become important for new users.

### Conclusions

The results of this study in terms of reproducibility are consistent with those previously described and the average skin temperature differences are similar as well. This consistency, as also noted by Owens et al., indicates that the changes seen in properly performed thermal scans are most likely due to actual physiological changes rather than equipment or technical error [19]. The results of this study add further evidence that paraspinal thermal scanning is a reliable method.
